# The Relationship between Family Meals and Mental Health Problems in Japanese Elementary School Children: A Cross-Sectional Study

**DOI:** 10.3390/ijerph18179281

**Published:** 2021-09-02

**Authors:** Noriko Kameyama, Yukina Morimoto, Ayako Hashimoto, Hiroko Inoue, Ikuko Nagaya, Kozue Nakamura, Toshiko Kuwano

**Affiliations:** 1 Graduate School of Integrated Pharmaceutical and Nutritional Sciences, University of Shizuoka, Shizuoka 422-8526, Japan; kameyama@u-shizuoka-ken.ac.jp (N.K.); nutr.ed1@u-shizuoka-ken.ac.jp (Y.M.); 2 Department of Food and Nutrition, Faculty of Home Economics, Kyoto Women’s University, Kyoto 605-8501, Japan; hashimoa@kyoto-wu.ac.jp; 3 Department of Nutrition and Health Sciences, Faculty of Food and Nutritional Sciences, Toyo University, Tokyo 374-0193, Japan; inoue003@toyo.jp; 4 Department of Food and Nutrition, Gifu City Women’s College, Gifu 501-0192, Japan; nagaya@gifu-cwc.ac.jp; 5 Gifu City Health Center, Gifu 500-8701, Japan; kozuen8@gmail.com

**Keywords:** family meal, mental health, Strengths and Difficulties Questionnaire (SDQ), children, behavioral problems

## Abstract

The relative burden of mental health problems in children is increasing worldwide. Family meals have attracted attention as an effective modifiable factor for preventing children’s mental health problems. We examined the relationship between family meals and mental health problems in Japanese elementary schoolchildren. A cross-sectional, self-administered questionnaire survey was conducted with guardians of children aged 7 to 12 years in Gifu Prefecture, Japan. Frequency of family meals and with whom the child eats breakfast, lunch, and dinner were assessed separately for weekdays and weekends/holidays. Mental health was assessed using the Japanese version of the parent-reported Strengths and Difficulties Questionnaire. Multivariate adjusted odds ratios (ORs) for borderline/abnormal mental health status were calculated using logistic regression analysis. Of the 678 children, 24.9% had borderline/abnormal mental health status. Children eating breakfast with their family less than once a week (adjusted OR, 4.79; 95% confidence interval (CI), 1.51–15.25) and those eating weekend breakfast alone (adjusted OR, 3.61; 95% CI, 1.42–9.23) had a higher prevalence of borderline/abnormal mental health status compared to those eating breakfast seven times a week and weekend breakfast with their family, respectively. These results suggest that family meals, especially breakfast, might be positively associated with better mental health in children.

## 1. Introduction

The global disease burden attributable to mental disorders has risen in all countries, and mental health problems are among the most important worldwide issues [1,2]. Mental health problems are likely to become one of the main public health challenges, even in children and adolescents [3]. In particular, the prevalence of mental health problems among these two groups of people in 2020 has been reported to be 10–20% worldwide [4,5], and their relative burden is increasing and is expected to accelerate even more in the future [3,4]. A substantial proportion of all adult mental health problems develop in childhood or early adolescence [6,7,8,9]. Early intervention and prevention offer hope to avoid adult mental health problems in the future and improve personal well-being and productivity [4,9]. Identification of positive and negative factors affecting mental health can also inform early interventions that can reduce the burden of these disorders [4].

The risk factors associated with children’s mental health are remarkably diverse and complicated, including genetic background, problems with the physical health and nutritional status of the child, physical and mental health of the family, deficiencies in the psychosocial and educational environment, exposure to harmful substances and toxins, violence, and abuse or neglect [4]. Lifestyle behaviors are key factors for the prevention of mental health conditions, given that they are largely modifiable, unlike many other mental disorder risk factors. Diet is a key modifiable factor that potentially influences the development of the brain with long-term exposure [10]. In considering the relationship between diet and mental health, one of the common approaches is to examine the effects of individual nutrients; an alternative approach is to examine the effects of the whole diet quality and eating patterns [11]. Despite some contradictory results, cross-sectional and prospective studies have documented an association between unhealthy dietary patterns or consumption of low-quality diet and higher levels of depression or worse mental health in children and adolescents [11,12,13,14,15,16,17]. A prospective study in adolescents indicated that improvements in diet quality were mirrored by improvements in mental health over the follow-up period while deteriorating diet quality was associated with poorer psychological functioning [13]. Diet quality is important regarding mental health status early in life.

Family meals, that is, eating meals with one’s family, may play an important role in promoting healthy eating behaviors in children. Among eating behaviors, family meals have been attracting attention as a way to improve the quality of meals. The evidence regarding the dietary benefits of family meals is clear. Among children and adolescents, an increased frequency of family meals is reportedly positively associated with healthier dietary outcomes [18,19,20]. This is also expected to improve physical health, e.g., by reducing the odds of children and adolescents becoming overweight [20]. Furthermore, a relationship between family meals and mental health has also been reported in adolescents. A greater frequency of family meals is inversely associated with adolescents’ high-risk behavior patterns such as alcohol and substance use [21,22,23,24,25], depressive symptoms [23,24,25,26], emotional difficulties [26], risk of eating disorders [20,25], and suicide [23]. One study reported an association between a low frequency of having lunch or dinner with a parent or guardian and adolescents’ feelings of loneliness and trouble sleeping due to worries [27].

However, relatively few studies have examined children with respect to family meals and mental health. The studies conducted thus far have shown that frequent family meals are inversely associated with disordered eating as a psychosocial outcome in children [25]. In these studies, mental health has chiefly been assessed based on the symptoms of the child. It is difficult to assess mental health status objectively. Considering this difficulty, the Strengths and Difficulties Questionnaire (SDQ) is one of the most widely used, standardized, and well-validated brief questionnaires for assessing child mental health problems and has been specially developed for use in epidemiological studies. In the SDQ, an increase in the total difficulties score indicates an increased risk of mental health problems [28,29]. The SDQ allows us to objectively assess mental health status in a research setting. In some studies conducted in New Zealand and the United Kingdom, adolescents’ mental health status was assessed using the SDQ and was found to be associated with the frequency of family meals [26,30]. These associations are likely to vary with ethnicity and age and should be examined to confirm whether similar associations are found in other regions and at other ages among children.

Furthermore, despite the importance of the topic, there are no standardized definitions or response options for “family meals” [31]. Systematic reviews indicate great variability regarding the measurement of the frequency of family meals, each study’s question was worded differently, and the reference period varied; some studies enquired about the frequency of certain meals and the number of family members present for the meal [19,20,32]. Therefore, a multifaceted evaluation of family meals is necessary.

This study aimed to clarify the relationship between eating meals with family members and children’s mental health among Japanese elementary schoolchildren. The novelty of this study is that children’s mental health status was assessed objectively using the validated SDQ, and their family meals were examined regarding both frequency and with whom they habitually ate each meal separately. Our research objective was to identify whether family meals are associated with children’s better mental health; the hypothesis was that the frequency of family meals and with whom the child eats meals are positively related to children’s mental health status.

## 2. Methods

### 2.1. Study Design and Population

This study was a cross-sectional, self-administered questionnaire survey conducted in all public elementary schools in Yamagata City, Gifu Prefecture, Japan, between May and June 2015. The study subjects included 2nd- to 6th-grade students (aged 7–12 years). The questionnaire was distributed to the guardians of all students at nine elementary schools (1141) by homeroom teachers. Guardians who were mainly in charge of preparing meals for their children were requested to complete the questionnaire. The completed questionnaires were collected by homeroom teachers, and a total of 868 questionnaires were collected (response rate 76%).

This study was conducted following the guidelines of the Declaration of Helsinki and its amendments, and all procedures were approved by the ethics committee of the University of Shizuoka (No. 26-3). The study has been registered in the UMIN Clinical Trials Registry (UMIN000033146). A written explanation was provided to all participants, and responses to the questionnaire were regarded as consent to participate.

### 2.2. Questionnaire

#### 2.2.1. Family Meal Frequency

To assess family meal frequency during breakfast and dinner, the guardians were asked to report the number of days a week in response to the question, “How many days per week does your child eat breakfast (or dinner) with the family?” Based on the responses, the frequency was classified into four categories: less than once a week, 1–3 times a week, 4–6 times a week, and 7 times a week. Lunch was evaluated only on weekends and holidays since school lunches are served at school on weekdays. The frequency of eating lunch with the family was assessed by the response to the following question: “Does your child eat lunch with the family on weekends and holidays?” chosen from three categories, namely “eat together most of the time,” “eat together half the time,” and “eat together rarely.”

#### 2.2.2. Mealtime Environment

With whom the child eats breakfast and dinner was assessed based on the response to the following question: “With whom does your child eat breakfast (or dinner)?”. For breakfast and dinner, the guardians were asked to choose from the following six qualitative categories, separately for weekdays and weekends or holidays: “eat with the whole family,” “eat with the adults in the family,” “eat only with children in the family,” “eat alone (although the child wants to eat with the family),” “eat alone apart from the family,” and “others.” These answer categories distinguished between eating situations in which the adults eat together (“eat with the adults in the family”) or only the children eat together (“eat only with children in the family”), when the whole family is not together, but the child does not eat alone either. For analysis, “eat alone (although the child wants to eat with the family)” and “eat alone apart from the family” were recoded as “eat alone,” and “others” was excluded.

#### 2.2.3. Strengths and Difficulties Questionnaire

Children’s mental health status was measured using the Japanese version of the parent-reported SDQ for children aged 4 to 16 years [28,33]. The SDQ is a brief screening instrument used to assess the positive and negative aspects of children’s behavior. It consists of 25 items that are classified into five subscales, each containing five items: hyperactivity, emotional symptoms, conduct problems, peer problems, and prosocial behavior. Each item is rated on a three-point scale: “not true” = 0, “somewhat true” = 1, and “certainly true” = 2. Positively worded items were reverse-scored. Each subscale score ranged from 0 to 10. The total difficulties score was generated from the sum of the scores of the above four difficulty subscales, excluding the prosocial scale, ranging from 0 to 40. The total difficulties score was categorized as “normal (0 to 12 points),” “borderline (13 to 15 points),” and “abnormal (16 to 40 points)” according to cutoff points that had previously been reported in a community-based sample of Japanese children [33]. Children were categorized as having a mental health problem if their total difficulties score was at either the abnormal or borderline level.

#### 2.2.4. Other Variables

We collected information on the following variables through a questionnaire that was designed for this study: child’s age, gender, presence of medical history, parental educational achievement, and family structure.

Regarding medical history, the guardian was asked whether the child had ever been diagnosed with any disease by a physician, including asthma and allergic diseases, and those who answered any disease were considered to have a history of the disease. Regarding parental educational achievement, respondents were asked to choose from the following four categories to indicate the number of years they had spent in school since the first grade of an elementary school: “9 years or less”, “10 to 12 years”, “13 to 16 years”, or “17 years or more”. As for family structure, based on the responses to the question about the relationship of the family members living together, we classified them into four categories, “great-grandparent/grandparent/parent/child,” “grandparent/parent/child,” “parent/child,” and “others” focusing on the generation.

### 2.3. Statistical Analyses

For analysis, we excluded children with missing data regarding family meal frequency, mealtime environment, the SDQ, or other variables used (gender, age, medical history, parental educational achievement, or family structure).

To test for differences in total difficulties scores of the SDQ through characteristics of children, we performed the Mann–Whitney *U* test for two categories and the Kruskal–Wallis test for three or more categories.

The children were divided into three groups according to the total difficulties score of the SDQ classification: normal, borderline, and abnormal. The associations between SDQ categories and the characteristics of children, family meal frequency, and mealtime environment were analyzed using Pearson’s chi-square test or Fisher’s exact test; the latter was conducted when the cells with the expected frequency of less than 5 were present in more than 20% of all the cells. Adjusted standardized residuals of 1.96 or more were significantly more than the other frequencies, and those of −1.96 or less were significantly less than the other frequencies.

To examine the relationship between children’s behavioral problems and their family meal frequency and mealtime environment, the three classifications of total difficulties score of the SDQ were combined into two groups: “normal” and “borderline/abnormal.” Logistic regression analysis was conducted to estimate odds ratios (ORs) and 95% confidence intervals (CIs) for being borderline/abnormal through family meal frequency and mealtime environment, with the highest frequency or “eat with the whole family” as the reference. Multivariate ORs were calculated by adjusting for the following potential confounders: gender (boy or girl), age (years), medical history (yes or no), family structure (great-grandparent/grandparent/parent/child, grandparent/parent/child, parent/child, or others), and parental educational achievement (≤9, 10–12, 13–16, or ≥17 years).

All statistical analyses were conducted using IBM SPSS version 25.0 J for Windows (IBM Japan, Ltd., Tokyo, Japan). Two-sided *p* values less than 0.05 were considered statistically significant.

## 3. Results

### 3.1. Characteristics of Participants

The total questionnaires distributed were 1141, of which 868 were collected (response rate: 76%). Some children were in more than one exclusion category; 190 children were excluded, and the final analysis comprised 678 children.

Respondents for the included children were 637 mothers (94%), 22 fathers (3.2%), 14 grandmothers (2.1%), 4 facility staff (0.6%), and 1 respondent who did not fill in any relation. The average age of the children was 9.3 ± 1.5 (mean ± SD) years old.

The characteristics of the 678 children are presented in Table 1. The guardians who studied up to high school (12 years or less) were 50.2%, and 49.7% studied up to junior college, university, or graduate school. Regarding family structure, nuclear families (parents and children) were the most common (65.8%), but 30% of the children lived with their grandparents.

### 3.2. Mental Health Status

The mean total difficulties score on the SDQ for all children was 9.5 ± 5.3 points. When the children were classified into three mental health status groups according to the total difficulties score on the SDQ, the prevalence values of each category were 75.1% for normal, 11.5% for borderline, and 13.4% for abnormal. The total difficulties score on the SDQ in boys averaged 10.2 ± 5.6 points, a value higher than that in girls (8.8 ± 5.0 points) (*p* = 0.002). Furthermore, the percentage of abnormalities was higher in boys than in girls (*p* = 0.008) (Table 1). No differences were observed in the distribution of mental health status for other background information of the children, such as their age, medical history, parental educational achievement, and family structure.

### 3.3. Family Meal Frequency and Mealtime Environment

The family meal frequency and mealtime environment of all subjects are presented in Table 2. Among all the children, 71.5% ate breakfast with their family seven times a week, while the next highest frequency was one to three times a week (16.1%). In contrast, 91.7% of children ate lunch on weekends and holidays with their families most of the time and more than 90% ate dinner with their family seven times a week. Regarding the frequency of family meals, less than 1% of the children rarely ate lunch and it was less than once a week for dinner, indicating that most of the children ate lunch and dinner with their families.

Conversely, the percentage of “eat with the whole family” in response to the question about who children eat breakfast and dinner with on weekdays and weekends was lower than that of “7 times a week” in response to the frequency of eating breakfast or dinner with the family. On weekdays, only 20.4% of children ate breakfast with their family, 29.9% ate breakfast with only children in the family, and 5.8% ate breakfast alone. On weekends, 39.7% of children ate breakfast with their family, which is higher than on weekdays, but 16.8% ate breakfast alone or with only children in the family. For dinner, many children ate with their families, 46.3% and 79.6% on weekdays and weekends, respectively, and more than 90% of children ate with the family or with adults. For dinner, less than 1% of children ate alone.

### 3.4. Relationship between Family Meal Frequency or Mealtime Environment and Mental Health Problems

Table 2 presents the family meal frequency and mealtime environment according to the degree of mental health problems. No relationships were identified between family meal frequency and mental health problems for breakfast, lunch, and dinner. Regarding the mealtime environment, a significant association was identified between weekend breakfast and mental health problems, that is, those who ate alone had a higher percentage of abnormal status, while those who ate with the family had a lower percentage of abnormal status and a higher percentage of normal status (*p* = 0.023).

As presented in Table 3, compared with the children who ate breakfast with their family seven times a week, those who ate breakfast with their family less than once a week had a significantly higher prevalence of borderline/abnormal mental health status (OR 3.93 (95% CI, 1.29−11.94), *p* = 0.016), and even after adjustment for potential confounders, it was still higher (adjusted OR 4.79 (95% CI, 1.51−15.25), *p* = 0.008).

Regarding the mealtime environment, weekend breakfast consumption was significantly associated with mental health status (Table 4). Compared to those who ate weekend breakfast with their family, those who ate breakfast alone had a higher OR of 2.69 (95% CI, 1.10−6.63, *p* = 0.031) for borderline/abnormal mental health problems, and even after adjustment, the OR was higher at 3.61 (95% CI, 1.42−9.23, *p* = 0.007). The ORs tended to be higher for those who ate with someone else in their family (with adults or children only). For breakfasts on weekdays and dinners on weekdays and weekends, no relationships were identified between mealtime environment and the risk of mental health problems.

## 4. Discussion

This study aimed to determine the relationship between family meals and mental health status as assessed objectively by the SDQ in Japanese elementary schoolchildren. It revealed the potential relationships between family meals and mental health status in Japanese elementary schoolchildren. We demonstrated that family breakfasts were related to the mental health of children, that is, children who ate breakfast with their family less frequently or those who ate breakfast alone on weekends and holidays had a higher frequency of mental health problems. Conversely, we did not identify a statistically significant association between family dinner and mental health status.

This is in line with other studies that have been conducted on adolescents and indicates a relationship between family meals and mental health. One of the unique features of this study is the association between mental health status and family meal status at breakfast, which has rarely been investigated in the past. However, this study found no significant association between the frequency of family dinner or with whom they ate dinner, and the mental health status of the children, even though these associations have been reported in adolescents [21,22,24]. One possible reason for this inconsistency is that the frequency of family dinner among the children in this study is relatively high compared to that in earlier studies among adolescents in Western countries, and most of the children ate dinner with their families. Although family dinner frequency was defined differently across studies, in earlier studies, about 15–20% of adolescents in Western countries had family dinner in the range of “never” to 0–2 days a week [21,22,24], while only less than 5% of the children in this study had family dinner less than three days a week. Furthermore, the family meal status of the children in this study was considered favorable compared to the results of a nationwide survey of 5th-grade students in Japan, which indicated that 15.3% of children ate breakfast alone and 2.2% ate dinner alone [34]. The status of family meal is affected by the age of the target population, as well as regional and cultural differences and family structure. In this study, 30% of the households consisted of three or more generations, and this may be part of the reason for the relatively higher frequency of family meals and fewer children eating alone than in other surveys.

The variable definitions of family meals among studies are also one of the possibilities for inconsistent results among the studies. Previous studies have investigated the “frequency of eating meals with family members” without limiting meals or limited to specific meals such as dinner. The definition of “with family” also varies, including most, all, and at least one parent [19,20,31,32]. Different definitions make direct comparisons difficult and require the standardization of survey methods. Therefore, in this study, we evaluated the status of family meals in terms of frequency and with whom children eat breakfast and dinner. The results indicated that the frequency of eating together seven days a week was 71.5% for breakfast and 91.9% for dinner; however, when asked with whom the children were eating, the percentage of eating with their family was lower than the frequency response. Both questions also confirmed the association between family breakfasts and mental health status in Japanese children. Not only the frequency but also the mealtime environment is useful for assessing the status of family meals. Establishing a validated method to assess the status of family meals is required.

Mental health assessment is difficult, and a variety of measures of mental health status have been used in studies of adolescents, including depressive symptoms, disordered eating, or problem behaviors such as substance use. We used the standardized and well-validated brief SDQ for children’s mental health assessment, which is an appropriate tool for measuring psychological difficulties in children, as it also captures issues less serious than diagnosed mental health problems, but the results can predict risk for the development of more serious problems [28,29]. We used cutoffs suggested by Matsuishi et al., in which approximately 10% of the general population are classified as “abnormal,” and 10% as “borderline,” as originally designed [28,33]. In this study, when the children were classified into three categories using this cutoff, the prevalence of each category was 75.1% for normal, 11.5% for borderline, and 13.4% for abnormal, with a slightly smaller percentage of normal and a higher percentage of borderline and abnormal. As for gender effects, boys had higher levels of difficulty than girls did. Such gender differences in SDQ scores are consistent with previous studies on the SDQ [33,35,36].

Utter et al. reported the association between frequency of family meals and mental health status assessed using the SDQ in adolescents aged 13 to 17 years [26]. The study reported that adolescents who ate with their families frequently had lower scores on the total difficulties score of the SDQ compared to those who rarely ate with their families [26]. Furthermore, family activity variables (such as going to other places and watching TV or videos) explained some of the association between mental health assessed by the SDQ, among which the most consistent association was observed in the frequency of eating a meal together for almost all ethnic groups [30]. The results in children indicated the same trend as these studies using the SDQ for assessing mental health problems. Since a small increase in the SDQ total difficulties score indicated an increased risk of mental health problems [29], family meals may prevent the development of mental health problems in children.

The mechanisms by which shared family meals are related to positive outcomes of mental health have not yet been empirically revealed; however, several possibilities have been suggested. Diet quality may be related to mental health status through brain development in children [10]. A cross-sectional study indicated that nutritional inadequacy plays an important role in mental health and may contribute to the pathogenesis of depression in schoolchildren aged 6–9 years [16]. Since family meal frequency is clearly and positively associated with dietary quality among children and adolescents [18,19,20], increasing the frequency of family meals is presumed to be related to children’s mental health status through diet quality. Furthermore, family meals also provide opportunities for communication, sharing of values, family connections, and parental monitoring [37,38]. Family mealtime communication was reportedly significantly associated with higher positive affect and engagement, and lower negative affect and stress [39]. Communication and other family functions during family meals are beneficial to children’s mental health. Any influence of diet on mental health is likely to be difficult to detect in the presence of a large number of potentially more powerful detrimental social, behavioral, and environmental factors [4,17]. The prevention of mental health problems in children contributes to the prevention of the onset of mental health problems in adults, suggesting that promoting family meals as a modifiable factor may be effective in preventing such problems.

Our study has several limitations that should be considered when interpreting the findings. First, since this study was conducted in a specific city in Japan, the generalization of the results must be limited. An investigation with a large population-based sample of children would provide additional valuable evidence to confirm the results of our study. Second, the self-administered questionnaires by the guardians may have over or underestimated the family meal status and children’s mental health status according to social desirability bias. Third, we were not able to investigate mealtime communication with family members and dietary intake, both of which have been reported to be associated with children’s mental health status. Fourth, among the subjects of this study, some of the subgroups, such as those who “eat alone” and those with a family meal frequency of less than once a week, showed extremely low observation values, and the results are not sufficiently robust. A survey with a larger number of participants is necessary. Finally, due to the cross-sectional study design, we cannot eliminate the possibility of reverse causality or a bidirectional effect, and we cannot conclude a causal relationship, that is, whether less family meal frequency affects mental health problems or mental health problems affect participation in family meals. Further studies are required to evaluate these mechanisms.

While we acknowledge these limitations, the strength of this study is that it is a complete survey of all elementary schoolchildren in a specific city. Therefore, we could evaluate the healthy general population, and the sampling error caused by extracting only a part of the target can be minimized. Moreover, this is the first study to focus on the relationship between children’s mental health status assessed using the validated SDQ and family meals in Japanese elementary schoolchildren. Therefore, our findings provide potentially useful information for the prevention of mental health problems in children. The results suggest the necessity to evaluate each meal and with whom the children are eating meals to understand the comprehensive status of family meals. Educational and public health initiatives aimed at promoting shared family meals may contribute not only to children’s physical health but also to their mental health.

## 5. Conclusions

Among Japanese elementary schoolchildren, we identified that those who eat breakfast with their family infrequently and those who eat breakfast alone on weekends and holidays were more likely to have worse mental health status assessed by the SDQ. This suggests that family meals, especially breakfast, might be positively associated with better mental health in children. Family meals may contribute to the improvement of children’s mental health.

## Figures and Tables

**Table 1:** Characteristics of Japanese elementary schoolchildren according to classification of mental health status assessed by the Strengths and Difficulties Questionnaire (N = 678).

	Total		Mental Health Status Assessed by SDQ						
			Normal		Borderline		Abnormal		*p*
	(N = 678)		(*n* = 509)		(*n* = 78)		(*n* = 91)		
Gender									
Boy	329	(48.5%)	235	(46.2%) ^‡^	36	(46.2%)	58	(63.7%) ^†^	0.008 ^φ^
Girl	349	(51.5%)	274	(53.8%) ^†^	42	(53.8%)	33	(36.3%) ^‡^	
Age (years)									
7	102	(15.0%)	72	(14.1%)	16	(20.5%)	14	(15.4%)	0.177 ^φ^
8	124	(18.3%)	90	(17.7%)	18	(23.1%)	16	(17.6%)	
9	148	(21.8%)	109	(21.4%)	11	(14.1%)	28	(30.8%) ^†^	
10	118	(17.4%)	95	(18.7%)	9	(11.5%)	14	(15.4%)	
11	167	(24.6%)	130	(25.5%)	20	(25.6%)	17	(18.7%)	
12	19	(2.8%)	13	(2.6%)	4	(5.1%)	2	(2.2%)	
Medical history									
Yes	582	(85.8%)	432	(84.9%)	67	(85.9%)	83	(91.2%)	0.279 ^φ^
No	96	(14.2%)	77	(15.1%)	11	(14.1%)	8	(8.8%)	
Parental educational achievement									
≤9 years	60	(8.8%)	43	(8.4%)	10	(12.8%)	7	(7.7%)	0.066 ^φ^
10–12 years	281	(41.4%)	200	(39.3%) ^‡^	30	(38.5%)	51	(56.0%) ^†^	
13–16 years	312	(46.0%)	248	(48.7%) ^†^	34	(43.6%)	30	(33.0%) ^‡^	
≥17 years	25	(3.7%)	18	(3.5%)	4	(5.1%)	3	(3.3%)	
Family structure									
Great-grandparent/grandparent/parent/child	20	(2.9%)	16	(3.1%)	2	(2.6%)	2	(2.2%)	0.236 ^§^
Grandparent/parent/child	197	(29.1%)	148	(29.1%)	25	(32.1%)	24	(26.4%)	
Parent/child	446	(65.8%)	337	(66.2%)	50	(64.1%)	59	(64.8%)	
Others	15	(2.2%)	8	(1.6%) ^‡^	1	(1.3%)	6	(6.6%) ^†^	

SDQ, Strengths and Difficulties Questionnaire. Values are numbers and percentages are in parentheses. ^φ^ Pearson’s chi-square test, ^§^ Fisher’s exact test. ^†^ Adjusted standardized residual ≥1.96; ^‡^ adjusted standardized residual ≤−1.96. Total difficulties score of SDQ classified 0–12 points as normal, 13–15 points as borderline, and 16–40 points as abnormal.

**Table 2:** Family meal frequency and mealtime environment for Japanese elementary schoolchildren according to the classification of mental health status assessed by the Strengths and Difficulties Questionnaire (N = 678).

	Total		Mental Health Status Assessed by SDQ						
			Normal		Borderline		Abnormal		*p*
	(N = 678)		(*n* = 509)		(*n* = 78)		(*n* = 91)		
Frequency of eating breakfast with their family									
7 times/week	485	(71.5%)	374	(73.5%)	53	(67.9%)	58	(63.7%)	0.177 ^φ^
4–6 times/week	71	(10.5%)	49	(9.6%)	9	(11.5%)	13	(14.3%)	
1–3 times/week	109	(16.1%)	80	(15.7%)	13	(16.7%)	16	(17.6%)	
<1 times/week	13	(1.9%)	6	(1.2%) ^‡^	3	(3.8%)	4	(4.4%)	
Frequency of eating lunch with their family on weekends and holidays									
most of the time	621	(91.7%)	469	(92.3%)	71	(91.0%)	81	(89.0%)	0.622 ^φ^
half the time	50	(7.4%)	35	(6.9%)	6	(7.7%)	9	(9.9%)	
rarely	6	(0.9%)	4	(0.8%)	1	(1.3%)	1	(1.1%)	
Frequency of eating dinner with their family									
7 times/week	623	(91.9%)	467	(91.7%)	73	(93.6%)	83	(91.2%)	0.965 ^§^
4–6 times/week	27	(4.0%)	20	(3.9%)	2	(2.6%)	5	(5.5%)	
1–3 times/week	25	(3.7%)	19	(3.7%)	3	(3.8%)	3	(3.3%)	
<1 times/week	3	(0.4%)	3	(0.6%)	0	(0.0%)	0	(0.0%)	
Breakfast environment on weekdays									
Eat with the whole family	138	(20.4%)	112	(22.0%)	10	(12.8%)	16	(17.6%)	0.587 ^φ^
Eat with the adults in the family	298	(44.0%)	220	(43.2%)	38	(48.7%)	40	(44.0%)	
Eat only with children in the family	203	(29.9%)	150	(29.5%)	25	(32.1%)	28	(30.8%)	
Eat alone	39	(5.8%)	27	(5.3%)	5	(6.4%)	7	(7.7%)	
Breakfast environment on weekends and holidays									
Eat with the whole family	269	(39.7%)	214	(42.0%) ^†^	32	(41.0%)	23	(25.3%) ^‡^	0.023 ^φ^
Eat with the adults in the family	295	(43.5%)	217	(42.6%)	31	(39.7%)	47	(51.6%)	
Eat only with children in the family	92	(13.6%)	65	(12.8%)	13	(16.7%)	14	(15.4%)	
Eat alone	22	(3.2%)	13	(2.6%)	2	(2.6%)	7	(7.7%) ^†^	
Dinner environment on weekdays									
Eat with the whole family	314	(46.3%)	243	(47.7%)	28	(35.9%) ^‡^	43	(47.3%)	0.073 ^§^
Eat with the adults in the family	322	(47.5%)	234	(46.0%)	42	(53.8%)	46	(50.5%)	
Eat only with children in the family	38	(5.6%)	29	(5.7%)	8	(10.3%)	1	(1.1%) ^‡^	
Eat alone	4	(0.6%)	3	(0.6%)	0	(0.0%)	1	(1.1%)	
Dinner environment on weekends and holidays									
Eat with the whole family	540	(79.6%)	410	(80.6%)	60	(76.9%)	70	(76.9%)	0.068 ^§^
Eat with the adults in the family	125	(18.4%)	91	(17.9%)	13	(16.7%)	21	(23.1%)	
Eat only with children in the family	12	(1.8%)	7	(1.4%)	5	(6.4%) ^†^	0	(0.0%)	
Eat alone	1	(0.1%)	1	(0.2%)	0	(0.0%)	0	(0.0%)	

SDQ, Strengths and Difficulties Questionnaire. Values are numbers and percentages are in parentheses. ^φ^ Pearson’s chi-square test, ^§^ Fisher’s exact test. ^†^ Adjusted standardized residual ≥1.96; ^‡^ adjusted standardized residual ≤−1.96. Total difficulties score of SDQ classified 0–12 points as normal, 13–15 points as borderline, and 16–40 points as abnormal.

**Table 3:** Relationship of the frequency of family meals and the prevalence of borderline/abnormal mental health status assessed by the Strengths and Difficulties Questionnaire among Japanese elementary schoolchildren (N = 678).

		Risk of Borderline/Abnormal Mental Health Status					
	*n*	Crude OR (95% CI)		*p*	Adjusted OR (95% CI)		*p*
Frequency of eating breakfast with their family							
7 times/week	485	1.00			1.00		
4–6 times/week	71	1.51	(0.88–2.61)	0.137	1.54	(0.87–2.73)	0.134
1–3 times/week	109	1.22	(0.76–1.96)	0.409	1.27	(0.78–2.07)	0.339
<1 times/week	13	3.93	(1.29–11.94)	0.016	4.79	(1.51–15.25)	0.008
Frequency of eating lunch with their family on weekends and holidays							
Most of the time	621	1.00			1.00		
Half the time	50	1.32	(0.70–2.49)	0.386	1.43	(0.74–2.78)	0.288
Rarely	6	1.54	(0.28–8.51)	0.619	1.56	(0.27–8.88)	0.618
Frequency of eating dinner with their family							
7 times/week	623	1.00			1.00		
4–6 times/week	27	1.05	(0.43–2.53)	0.917	0.88	(0.36–2.19)	0.791
1–3 times/week	25	0.95	(0.37–2.41)	0.906	0.92	(0.35–2.39)	0.860
<1 times/week	3	—	—	—	—	—	—

CI, confidence interval; OR, odds ratio; SDQ, Strengths and Difficulties Questionnaire. Values are ORs and 95% CIs are in parentheses for borderline/abnormal (total difficulties score of SDQ is 13 to 40 points) against normal mental health status (total difficulties score of SDQ is 0 to 12 points). Adjusted ORs were adjusted for gender (boy or girl), age (years), presence of medical history (yes or no), family structure (great-grandparent/grandparent/parent/child, grandparent/parent/child, parent/child, or others), and parental educational achievement (≤9, 10–12, 13–16, or ≥17 years).

**Table 4:** Relationship of mealtime environment and prevalence of borderline/abnormal mental health status assessed by the Strengths and Difficulties Questionnaire among Japanese elementary schoolchildren (N = 678).

		Risk of Borderline/Abnormal Mental Health Status					
	*n*	Crude OR (95% CI)		*p*	Adjusted OR (95% CI)		*p*
Breakfast environment on weekdays							
Eat with the whole family	138	1.00			1.00		
Eat with the adults in the family	298	1.53	(0.93–2.51)	0.096	1.63	(0.97–2.73)	0.065
Eat only with children in the family	203	1.52	(0.90–2.58)	0.120	1.61	(0.93–2.78)	0.089
Eat alone	39	1.91	(0.86–4.27)	0.113	2.03	(0.89–4.62)	0.090
Breakfast environment on weekends and holidays							
Eat with the whole family	269	1.00			1.00		
Eat with the adults in the family	295	1.40	(0.94–2.07)	0.095	1.46	(0.97–2.20)	0.068
Eat only with children in the family	92	1.62	(0.94–2.77)	0.080	1.67	(0.96–2.92)	0.072
Eat alone	22	2.69	(1.10–6.63)	0.031	3.61	(1.42–9.23)	0.007
Dinner environment on weekdays							
Eat with the whole family	314	1.00			1.00		
Eat with the adults in the family	322	1.29	(0.90–1.85)	0.170	1.40	(0.97–2.04)	0.076
Eat only with children in the family	38	1.06	(0.48–2.35)	0.882	1.21	(0.54–2.74)	0.643
Eat alone	4	1.14	(0.12–11.14)	0.910	1.12	(0.10–12.01)	0.928
Dinner environment on weekends and holidays							
Eat with the whole family	540	1.00			1.00		
Eat with the adults in the family	125	1.18	(0.76–1.83)	0.465	1.24	(0.79–1.95)	0.359
Eat only with children in the family	12	2.25	(0.70–7.22)	0.172	2.61	(0.78–8.70)	0.119
Eat alone	1	—	—	—	—	—	—

CI, confidence interval; OR, odds ratio; SDQ, Strengths and Difficulties Questionnaire. Values are ORs and 95% CIs are in parentheses for borderline/abnormal (total difficulties score of SDQ is 13 to 40 points) against normal mental health status (total difficulties score of SDQ is 0 to 12 points). Adjusted ORs were adjusted for gender (boy or girl), age (years), presence of medical history (yes or no), family structure (great-grandparent/grandparent/parent/child, grandparent/parent/child, parent/child, or others), and parental educational achievement (≤9, 10–12, 13–16, or ≥17.

## Data Availability

The datasets used and analyzed during the current study are available from the corresponding author upon reasonable request.

## Abbreviations

CI	confidence interval
OR	odds ratio
SD	standard deviation
SDQ	Strengths and Difficulties Questionnaire

## References

[1] Patel V., Saxena S., Lund C., Thornicroft G., Baingana F., Bolton P., Chisholm D., Collins P.Y., Cooper J.L., Eaton J. (2018). The Lancet Commission on global mental health and sustainable development. Lancet.

[2] Rehm J., Shield K.D. (2019). Global Burden of Disease and the Impact of Mental and Addictive Disorders. Curr. Psychiatry Rep..

[3] Baranne M.L., Falissard B. (2018). Global burden of mental disorders among children aged 5–14 years. Child Adolesc. Psychiatry Ment. Health.

[4] Kieling C., Baker-Henningham H., Belfer M., Conti G., Ertem I., Omigbodun O., Rohde L.A., Srinath S., Ulkuer N., Rahman A. (2011). Child and adolescent mental health worldwide: Evidence for action. Lancet.

[5] Wolrd Health Organization (2020). Adolescent Mental Health. https://www.who.int/news-room/fact-sheets/detail/adolescent-mental-health.

[6] Kessler R.C., Angermeyer M., Anthony J.C., Graaf R.D.E., Demyttenaere K., Gasquet I., Girolamo G.D., Gluzman S., Gureje O., Haro J.A. (2007). Lifetime prevalence and age-of-onset distributions of mental disorders in the World Health Organization’s World Mental Health Survey Initiative. World Psychiatry.

[7] Kim-Cohen J., Caspi A., Moffitt T.E., Harrington H., Milne B.J., Poulton R. (2003). Prior juvenile diagnoses in adults with mental disorder: Developmental follow-back of a prospective-longitudinal cohort. Arch. Gen. Psychiatry.

[8] Kessler R.C., Berglund P., Demler O., Jin R., Merikangas K.R., Walters E.E. (2005). Lifetime Prevalence and Age-of-Onset Distributions of DSM-IV Disorders in the National Comorbidity Survey Replication. Arch. Gen. Psychiatry.

[9] Kessler R.C., Amminger G.P., Aguilar-Gaxiola S., Alonso J., Lee S., Ustun T.B. (2007). Age of onset of mental disorders: A review of recent literature. Curr. Opin. Psychiatry.

[10] Bryan J., Osendarp S., Hughes D., Calvaresi E., Baghurst K., Van Klinken J.-W. (2004). Nutrients for Cognitive Development in School-aged Children. Nutr. Rev..

[11] Khalid S., Williams C., Reynolds S.A. (2016). Is there an association between diet and depression in children and adolescents? A systematic review. Br. J. Nutr..

[12] O’Neil A., Quirk S., Housden S., Brennan-Olsen S., Williams L., Pasco J.A., Berk M., Jacka F.N. (2014). Relationship Between Diet and Mental Health in Children and Adolescents: A Systematic Review. Am. J. Public Health.

[13] Jacka F.N., Kremer P., Berk M., De Silva-Sanigorski A.M., Moodie M., Leslie E., Pasco J.A., Swinburn B.A. (2011). A Prospective Study of Diet Quality and Mental Health in Adolescents. PLoS ONE.

[14] Hoare E., Werneck A.O., Stubbs B., Firth J., Collins S., Corder K., Van Sluijs E.M.F. (2020). Association of Child and Adolescent Mental Health with Adolescent Health Behaviors in the UK Millennium Cohort. JAMA Netw. Open.

[15] Wiles N.J., Northstone K., Emmett P., Lewis G. (2007). ‘Junk food’ diet and childhood behavioural problems: Results from the ALSPAC cohort. Eur. J. Clin. Nutr..

[16] Rubio-López N., Morales-Suárez-Varela M., Pico Y., Livianos-Aldana L., Llopis-González A. (2016). Nutrient Intake and Depression Symptoms in Spanish Children: The ANIVA Study. Int. J. Environ. Res. Public Health.

[17] Jacka F.N., Rothon C., Taylor S., Berk M., Stansfeld S.A. (2012). Diet quality and mental health problems in adolescents from East London: A prospective study. Soc. Psychiatry Psychiatr. Epidemiol..

[18] Woodruff S.J., Hanning R.M., McGoldrick K., Brown K.S. (2010). Healthy eating index-C is positively associated with family dinner frequency among students in grades 6–8 from Southern Ontario, Canada. Eur. J. Clin. Nutr..

[19] Fulkerson J.A., Larson N., Horning M., Neumark-Sztainer D. (2014). A review of associations between family or shared meal frequency and dietary and weight status outcomes across the lifespan. J. Nutr. Educ. Behav..

[20] Hammons A.J., Fiese B.H. (2011). Is frequency of shared family meals related to the nutritional health of children and adolescents?. Pediatrics.

[21] Musick K., Meier A. (2012). Assessing Causality and Persistence in Associations Between Family Dinners and Adolescent Well-Being. J. Marriage Fam..

[22] Fulkerson J.A., Story M., Mellin A., Leffert N., Neumark-Sztainer D., French S.A. (2006). Family Dinner Meal Frequency and Adolescent Development: Relationships with Developmental Assets and High-Risk Behaviors. J. Adolesc. Health.

[23] Eisenberg M.E., Olson R.E., Neumark-Sztainer D., Story M., Bearinger L.H. (2004). Correlations Between Family Meals and Psychosocial Well-being Among Adolescents. Arch. Pediatr. Adolesc. Med..

[24] Fulkerson J.A., Kubik M.Y., Story M., Lytle L., Arcan C. (2009). Are there nutritional and other benefits associated with family meals among at-risk youth?. J. Adolesc. Health.

[25] Harrison M.E., Norris M.L., Obeid N., Fu M., Weinstangel H., Sampson M. (2015). Systematic review of the effects of family meal frequency on psychosocial outcomes in youth. Can. Fam. Physician.

[26] Utter J., Denny S., Peiris-John R., Moselen E., Dyson B., Clark T. (2017). Family Meals and Adolescent Emotional Well-Being: Findings from a National Study. J. Nutr. Educ. Behav..

[27] Escobar D.F.S.S., De Jesus T.F., Noll P.R.E.S., Noll M. (2020). Family and School Context: Effects on the Mental Health of Brazilian Students. Int. J. Environ. Res. Public Health.

[28] Goodman R. (1997). The Strengths and Difficulties Questionnaire: A Research Note. J. Child Psychol. Psychiatry.

[29] Goodman A., Goodman R. (2009). Strengths and Difficulties Questionnaire as a Dimensional Measure of Child Mental Health. J. Am. Acad. Child Adolesc. Psychiatry.

[30] Maynard M.J., Harding S. (2009). Ethnic differences in psychological well-being in adolescence in the context of time spent in family activities. Soc. Psychiatry Psychiatr. Epidemiol..

[31] Takimoto H., Sarukura N., Ishikawa-Takata K. (2015). How to define family meals in “Shokuiku” (Food and Nutrition Education). J. Nutr. Sci. Vitaminol..

[32] Valdés J., Rodríguez-Artalejo F., Aguilar L., Jaén-Casquero M.B., Royo-Bordonada M. (2012). Frequency of family meals and childhood overweight: A systematic review. Pediatr. Obes..

[33] Matsuishi T., Nagano M., Araki Y., Tanaka Y., Iwasaki M., Yamashita Y., Nagamitsu S., Iizuka C., Ohya T., Shibuya K. (2008). Scale properties of the Japanese version of the Strengths and Difficulties Questionnaire (SDQ): A study of infant and school children in community samples. Brain Dev..

[34] Japan Sport Council Report on the 2010 Survey on the Dietary Conditions of Children and Students. https://www.jpnsport.go.jp/anzen/school_lunch/tabid/1490/Default.aspx.

[35] Moriwaki A., Kamio Y. (2014). Normative data and psychometric properties of the strengths and difficulties questionnaire among Japanese school-aged children. Child Adolesc. Psychiatry Ment. Health.

[36] Shibata Y., Okada K., Fukumoto R., Nomura K. (2014). Psychometric properties of the parent and teacher forms of the Japanese version of the Strengths and Difficulties Questionnaire. Brain Dev..

[37] Utter J., Denny S., Robinson E., Fleming T., Ameratunga S., Grant S. (2013). Family meals and the well-being of adolescents. J. Paediatr. Child Health.

[38] Fulkerson J.A., Pasch K.E., Stigler M.H., Farbakhsh K., Perry C.L., Komro K.A. (2010). Longitudinal associations between family dinner and adolescent perceptions of parent–child communication among racially diverse urban youth. J. Fam. Psychol..

[39] Offer S. (2013). Assessing the relationship between family mealtime communication and adolescent emotional well-being using the experience sampling method. J. Adolesc..

